# Population Graph-Based Multi-Model Ensemble Method for Diagnosing Autism Spectrum Disorder

**DOI:** 10.3390/s20216001

**Published:** 2020-10-22

**Authors:** Zarina Rakhimberdina, Xin Liu, Tsuyoshi Murata

**Affiliations:** 1 Department of Computer Science, Tokyo Institute of Technology, Tokyo 152-8552, Japan; murata@c.titech.ac.jp; 2 AIST-Tokyo Tech Real World Big-Data Computation Open Innovation Laboratory, Tokyo Institute of Technology, Tokyo 152-8550, Japan; xin.liu@aist.go.jp; 3 AI Research Center, National Institute of Advanced Industrial Science and Technology, Tokyo 100-8921, Japan

**Keywords:** brain functional connectivity, graph neural network, graph signal processing

## Abstract

With the advancement of brain imaging techniques and a variety of machine learning methods, significant progress has been made in brain disorder diagnosis, in particular Autism Spectrum Disorder. The development of machine learning models that can differentiate between healthy subjects and patients is of great importance. Recently, graph neural networks have found increasing application in domains where the population’s structure is modeled as a graph. The application of graphs for analyzing brain imaging datasets helps to discover clusters of individuals with a specific diagnosis. However, the choice of the appropriate population graph becomes a challenge in practice, as no systematic way exists for defining it. To solve this problem, we propose a population graph-based multi-model ensemble, which improves the prediction, regardless of the choice of the underlying graph. First, we construct a set of population graphs using different combinations of imaging and phenotypic features and evaluate them using Graph Signal Processing tools. Subsequently, we utilize a neural network architecture to combine multiple graph-based models. The results demonstrate that the proposed model outperforms the state-of-the-art methods on Autism Brain Imaging Data Exchange (ABIDE) dataset.

## 1. Introduction

With the advent of neural networks, the analysis of complex brain imaging data, such as functional magnetic resonance imaging (fMRI) data, has become more feasible. Numerous applications of neural networks have been proposed, including those that are based on direct image processing of brain data [1,2] and those that utilize phenotypic information of subjects for disease prediction [3,4,5]. In this work, we focus on Autism Spectrum Disorders (ASD) prediction while using functional connectivity networks from resting-state fMRI (rs-fMRI).

Resting-state functional connectivity (RSFC) measures the temporal correlation between the blood-oxygen-level-dependent signal in different brain areas during a resting or task-negative state [6]. RSFC can reveal new patterns in the brain network that can lead to neurological disorders and thus are widely used to study brain organization and mental disorder [7,8,9]. RSFC is computed for each subject and it is represented while using a square matrix where each entry corresponds to the strength of the functional connectivity between two regions of the brain. Along with RSFC, fMRI datasets contain subject-level non-imaging phenotypic information. For example, the Autism Brain Imaging Data Exchange (ABIDE) database combines both imaging (RSFC) and phenotypic information, including sex, age, and imaging site [10]. Phenotypic information, in particular, sex, was shown to be useful for the prediction of ASD, since the disease affects females less frequently than males [11]. The efficient use of both imaging and non-imaging information for neurological disorder prediction has become the focus of many recent works [4,5,12,13].

### 1.1. Statistical Models and Deep Neural Networks Using Imaging Information

Many methods for studying brain disorders have been developed based on RSFC features alone, without using phenotypic features [12,14,15,16,17,18]. For example, [18] used RSFC and developed a multilayered convolutional neural network for predicting neurodevelopment. The authors in [12] presented a statistical kernel regression method that achieved comparable accuracies with Deep Neural Networks on behavior and demographics prediction tasks. With regard to ASD, [15] proposed an autoencoder-based deep neural network and achieved state-of-the-art classification accuracy on a subset of 964 subjects from the ABIDE dataset. The authors in [2] further improved the classification accuracy up to 70.22% while using convolutional neural networks.

### 1.2. Graphs and Graph Neural Networks Using Both Imaging and Non-Imaging Information

**Figure 1:**
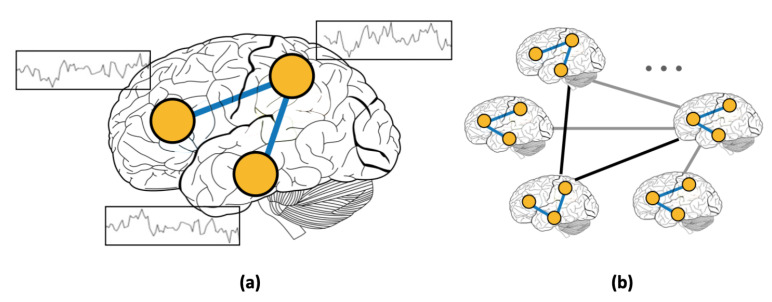
(**a**) Resting-state functional connectivity (RSFC) graph and (**b**) population graph.

In addition to using RSFC features, several studies focused on incorporating phenotypic information using graphs [4,19]. Graphs present a natural way of modeling complex interactions by combining features of different modalities [20,21,22]. For example, in social networks, nodes represent individuals, and the presence of a link between two nodes signifies the existence of a friendship between the corresponding individuals. Unlike social networks, where links are predefined, graphs that are constructed from medical data require a more elaborate choice of the subjects’ features for defining edges. In this work, we use graphs to represent inter-subject connectivity or population graph composed of the entire set of human subjects (Figure 1b). Each subject is modeled as a node with corresponding RSFC data, and each edge is defined based on the similarity between subjects’ features (RSFC, age, sex, etc.) [4]. Thus, the advantage of using graphs for medical diagnosis is multi-fold. First, modeling the subject’s data as a population graph allows for incorporating subjects’ non-imaging features [4,19]. Second, the rich set of analytical tools that were developed to infer complex patterns in graphs [23,24] allows for efficient computations on brain imaging datasets.

**Figure 2:**
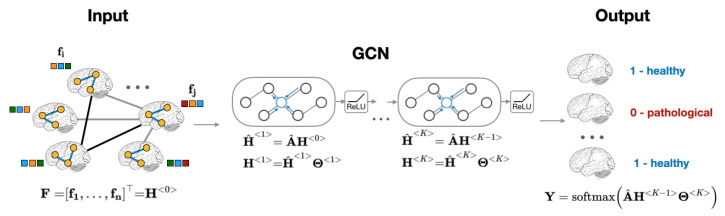
Overview of subject classification using graph neural networks. The model’s inputs are an adjacency matrix representation $\hat{A}$ of the population graph and a set of subjects’ RSFC features F.

Recently, Graph Convolutional Neural Network, or GCN, was introduced as an efficient method for node classification on graphs [23,24]. Unlike traditional methods that focus on either the features (regression models [12], convolutional neural networks [1], etc.) or on the network structure (community detection algorithms [25,26,27], node embeddings [28,29], etc.), GCN provides a computational framework that accounts for both node features and graph structure [23,30]. The aggregation of global neighborhood-level information is accomplished via graph convolution operation, which, in contrast to standard image convolutions, is performed by matrix multiplication of graph Laplacian [23]. Figure 2 presents the computational pipeline of GCN that is applied to brain imaging data.

Related works [4,31] showed graph-based models to be useful in improving classification performance on brain imaging data. In the study of Autism Spectrum Disorder and Alzheimer’s disease [4], authors defined edges in a population graph based on the similarity between phenotypic features and RSFC patterns of the subjects. To identify the most informative phenotypic features, the authors compared the performance of GCN on the set of population graphs that were constructed using different combinations of those features. Based on the results, the authors concluded that, along with RSFC, the sex of the subject and location of the imaging facility contributed significantly towards the improved prediction of Autism Spectrum Disorder. Whereas, for Alzheimer’s disease, a different combination of features (sex and genetic information) resulted in the best model’s accuracy. The authors in [31] proposed using a set of graphs with randomly removed edges to mitigate the problem of choosing the best performing graph. However, there is a significant problem with the previous works: a lack of systematic approach to choosing a particular graph definition over the other. In most cases, the similarity function that is used to define the edges becomes problem-specific, i.e., the choice of a particular graph definition might work for one dataset, but fail or not be applicable for others. Therefore, there is a need for a robust model that is less sensitive to the choice of the underlying graph construction method.

We address this problem by building a multi-model ensemble, which consists of two stages. In the first stage, we analyze all possible graph configurations and define a method for selecting the best performing graphs, i.e., the graphs that contribute towards improving the prediction accuracy. We find that merely combining all possible graph configurations does not help to improve the prediction accuracy. This is because some of the low-quality graphs carry noisy information and, thus, decrease the model’s performance. Therefore, we use tools from graph signal processing (GSP) [32,33] to analyze the properties of the constructed graphs and select the ones that contribute positively towards accuracy. We use GSP tools, such as graph Fourier Transform (GFT) and graph frequencies, to analyze RSFC signals that are defined on the nodes of the population graph. Decomposing a graph signal into low and high-frequency components allows us to compare different graphs structures in terms of signal smoothness. In particular, based on the characteristics of signal smoothness, we are interested in selecting the features that have a higher magnitude of contribution towards the graph signal. In the second stage, we select the best performing low-frequency graphs and propose a multi-model ensembling method that further improves the prediction accuracy. The use of GSP for feature selection on fMRI data and the proposed ensembling method are the novelties of this work that distinguish our method from the traditional ones.

Several ensembling schemes have been proposed in the literature with the goal of increasing the accuracy of the prediction in different domains by integrating multiple models. For example, [31] used a simple averaging of the probability outputs from individual models for ASD classification. The authors in [34] implemented a majority voting algorithm for combining different deep learning classifiers on text, images, and video. We propose an end-to-end deep neural network-based ensemble model that uses the weighting mechanism for assigning different levels of importance for each model’s prediction.

**Contribution:** In this work, we introduce a population graph-based multi-model ensemble method for brain disorder classification while using functional magnetic resonance imaging (fMRI) data. The contributions of this work are listed below:

- An evaluation of the graph spectrum of population graphs using tools from the field of GSP. Using frequency filtering, we improve the model’s accuracy and computational speed.
- An end-to-end deep neural network-based ensemble model with a weighting mechanism.
- State-of-the-art performance on the ABIDE dataset. Our results show that using both graph signal filtering and an end-to-end ensemble method leads to improved classification accuracy, resulting in a state-of-the-art classification accuracy of 73.13% accuracy for the ABIDE dataset (2.91% improvement as compared to the best result reported in related works [2]).

The proceeding content of this paper is organized, as follows. First, we describe the fMRI datasets that were used in the analysis in Section 2.1. In Section 2.2, we introduce the process of population graph construction using fMRI data and describe the differences between various edge defining functions. In Section 2.3, we introduce the basic concepts from GSP, including graph Fourier transform and filtering a graph signal into frequency components. Subsequently, we move on to analyzing multiple population graphs while using the tools from Graph Signal Processing in Section 2.4. Section 3 shows the detailed results of our proposed multi-model population graph-based ensemble. Finally, we discuss the results in Section 4.

## 2. Methods

Constructing a population graph from the set of patients and control subjects is not a straightforward task, as there exist multiple edge definitions that map the data to the graph structure. In a graph setting, after defining subjects as nodes, we are interested in identifying the features that capture the intrinsic relationship between the nodes. We consider the optimal graph structure to be the one in which clusters of patients and healthy subjects can be well separated. Learning the optimal graph structure becomes even more crucial in the later stage, as the most optimal graph topology permits subsequent efficient data processing. In the following section, we first describe the process of population graph construction that was adopted in the literature. We then proceed onto the analysis and selection of the best performing graphs using Graph Signal Processing (GSP) tools. Finally, we present the ensemble of multiple graph-based models on brain imaging data.

### 2.1. Dataset

In this section, we describe the publicly available fMRI dataset that was used in our experiments (see Table 1). Autism Brain Imaging Data Exchange (ABIDE) ( http://preprocessed-connectomes-project.org/abide/) dataset combines structural and functional MRI data of 1112 subjects from 17 international acquisition sites, which we refer to as imaging sites [10]. We use the subset of 871 subjects: 403 patients with Autism Spectrum Disorder (ASD) and 468 healthy individuals. Both imaging and non-imaging data are provided in the ABIDE. A single RSFC matrix represents the functional connectivity between 111 regions of interest (ROIs) in the brain extracted from the fMRI scan (we refer to [4] for more details on fMRI preprocessing). Non-imaging data correspond to phenotypic features, such as sex, age, and imaging site. We used imaging data in the form of RSFC matrices and non-imaging data that were defined by phenotypic measures in order to construct a population graph. Graph construction is discussed in more detail in Section 2.2.

### 2.2. Graph Construction

We describe the process of population graph construction adopted by [4]. Using the set of healthy controls and patients with ASD, we define the population graph, as follows. The graph nodes represent the subjects from the dataset, and edges connecting the nodes represent the similarity between subjects’ imaging and phenotypic features. More explicitly, we construct an undirected weighted graph G=(V,E,W), where the set of nodes $V=\{v_{1},\cdots ,v_{n}\}$ corresponds to a set of subjects. Each node $v_{i}$ is associated with a *d*-dimensional feature vector $x_{i}$ extracted from fMRI imaging data in the form of RSFC. The feature matrix $X\in R^{n\times d}$ consists of stacked feature vectors of *n* nodes in the graph. The set of edges E⊆V×V corresponds to links between the nodes, and W:E↦R is a function that assigns weight $w_{ij}$ to each edge, as follows:

(1) $$W\left(i,j\right)=\mathrm{sim}\left(x_{i},x_{j}\right)\sum_{h=1}^{H}1\left[M_{h}\left(v_{i}\right)=M_{h}\left(v_{j}\right)\right],$$

where $\mathrm{sim}\left(x_{i},x_{j}\right)$ is defined based on correlation distance between lower triangular elements of RSFC matrices, and $M_{h}\left(v_{i}\right)$ is categorical phenotypic feature value corresponding to node $v_{i}$. Categorical phenotypic features are sex (male/female), imaging site number, and age (a categorical value that corresponds to the age group).

From the above definition, it is clear that the parameter that affects the graph topology is the edge defining function W. Therefore, we briefly describe the variety of edge defining functions proposed in this and earlier works. For clarity, we categorize the resulting graphs into four groups:

1. *sim_RSFC*: a fully connected weighted graph constructed using correlation between RSFC features. This corresponds to using a weight function $W\left(i,j\right)=\mathrm{sim}\left(x_{i},x_{j}\right)$.
2. *sim_phenotype* graphs: graphs which are constructed using a combination of phenotypic features (*site, sex, age*). The graph construction corresponds to taking only the second part of the Equation (1), i.e when $W\left(i,j\right)=\sum_{h=1}^{H}1\left[M_{h}\left(v_{i}\right)=M_{h}\left(v_{j}\right)\right]$. In total, there are seven *sim_phenotype* graphs constructed while using combinations of three phenotypic features: *sim_site, sim_age, sim_sex, sim_site_age, sim_site_sex, sim_sex_age*, and *sim_site_age_sex*.
3. *sim_RSFC_phenotype* graphs: graphs which utilize the combination of RSFC features and phenotypic features for edge definition. Similarly, there are seven *sim_RSFC_phenotype* graphs in total: *sim_RSFC_site, sim_RSFC_age, sim_RSFC_sex, sim_RSFC_site_age, sim_RSFC_site_sex, sim_RSFC_sex_age*, and *sim_RSFC_site_age_sex*. Note that when we are not using any features, the graph becomes *sim_RSFC*, which corresponds to the first group.
4. Baseline graphs: (a) *FC*—a fully connected graph with edge weight equal to 1, (b) *identity* graph—a fully disconnected graph with adjacency matrix equal to identity matrix, and (c) *random* graph, constructed by randomly assigning binary edges in *identity* graph.

### 2.3. Graph Signal Processing

After constructing multiple population graphs thhat are based on the different choice of edge defining function, we are interested in identifying the ones which capture the most optimal representation of the dataset. For this purpose, we utilize the tools from Graph Signal Processing (GSP) to analyze the underlying structures of the resulting graphs. This section describes the fundamental concepts of GSP, such as graph Fourier Transform and graph filtering. GSP tools help to perform a quantitative comparison between graphs that are produced using a different choice of edge definition. In particular, based on the characteristics of signal smoothness, we are interested in selecting the most relevant features for population graphs constructed using different edge defining functions.

#### 2.3.1. Graph Fourier Transform

GSP, in contrast to classical signal processing, analyzes signals that reside on the nodes of graphs. These graph signals can also be referred to as node features. With GSP, we can generalize the signal processing concepts, such as signal smoothness and signal filtering, to a graph domain. We introduce the fundamental concepts of GSP while using conventional definitions of the adjacency matrix, graph Laplacian matrix, and graph Fourier transform [35].

Given a graph G with the adjacency matrix A and degree matrix D, the combinatorial Laplacian matrix of graph G is defined as a difference $L=D-A\in R^{n\times n}$. A feature vector $x\in R^{d}$ that is defined on each node is called graph signal. Because L is a positive semidefinite matrix, it can be decomposed into a complete set of orthonormal eigenvectors and corresponding eigenvalues:

(2) $$L=U\Lambda U^{T},$$

where U is a matrix containing eigenvectors as columns and Λ is a diagonal matrix that contains eigenvalues along the diagonal.

Finally, we introduce the graph Fourier transform (GFT) and its inverse, which is used to represent a graph signal in node and spectral domains. Given a signal $x\in R^{d}$ and eigendecomposition of Laplacian L, the graph Fourier transform of x is defined as $\hat{x}=U^{⊤}x$ and represents the signal in the graph spectral domain. While the inverse graph Fourier transform of $\hat{x}$ is defined as $x=U\hat{x}$. $\hat{x}$ is also referred to as a frequency component of x.

**Figure 3:**
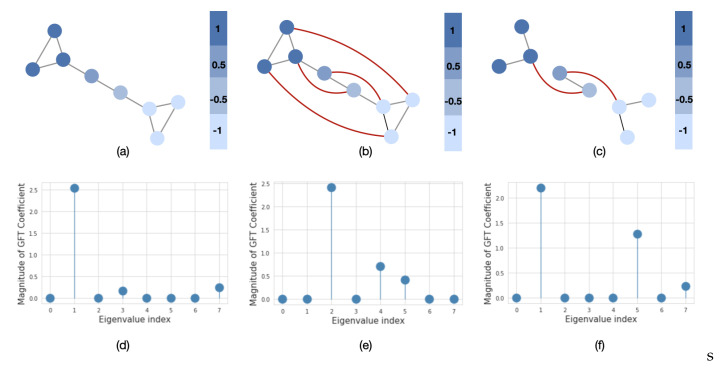
The importance of the graph structure. We plot the graph signal in the node (upper row) and spectral (lower row) domains. The red lines in (**b**) and (**c**) represent the addition of new edges to the original graph in (**a**). The eigenvalues in (**d**–**f**) are sorted in order of the increasing magnitude.

Similarly to time series analysis, the graph frequency components $\hat{x}$ represent the signal variation with respect to the graph structure. In order to evaluate how much signal varies, we use a graph’s spectral representation. For example, in Figure 3a, we plot a barbel graph with two clusters and a fixed signal over its nodes. The signal varies smoothly over the graph, since more similar values appear on neighboring nodes (low-frequency signals). The spectral representation of this graph in Figure 3d shows that the signal mainly consists of low-frequency components. However, if we rewire or remove some edges from the original graph, i.e., introduce dissimilar signals on neighboring nodes (Figure 3b,c), then we can observe that the signal will have more energy in the higher frequencies (Figure 3e,f). The last two graphs are referred to as high-frequency graphs. In population graph construction, we are particularly interested in the case when the graph is constructed in such a way that the neighboring nodes have similar features. This property of the graph is referred to as global smoothness [36].

#### 2.3.2. Graph Filtering

The knowledge about whether the graph is smooth or not helps to perform efficient computations by applying graph filtering [32]. It has been shown in [37] that smooth signals have compressible Fourier coefficients. This is because the sorted magnitude of Fourier coefficients exhibits power-law decay; thus, the largest coefficient can be used to approximate the signal. Using this fact, we can filter the original graph signal x by extracting the signal components corresponding to different frequencies, as follows:

(3) $$\tilde{x}={\mathrm{UHU}}^{⊤}x,$$

where H is a diagonal filter matrix. Specifically, we can define a low-pass filtering for *k* lowest graph frequencies, by setting the diagonal elements of H to 1 for first *k* eigenvalues and 0 otherwise. This is equivalent to only using first *k* eigenvectors:

(4) $${\tilde{x}}_{k}=U_{\left[0:k\right]}{\mathrm{HU}}_{\left[0:k\right]}^{⊤}x.$$

### 2.4. Analysis of Population Graphs Using GSP

#### 2.4.1. Evaluation of Fourier Transform Coefficients

**Figure 4:**
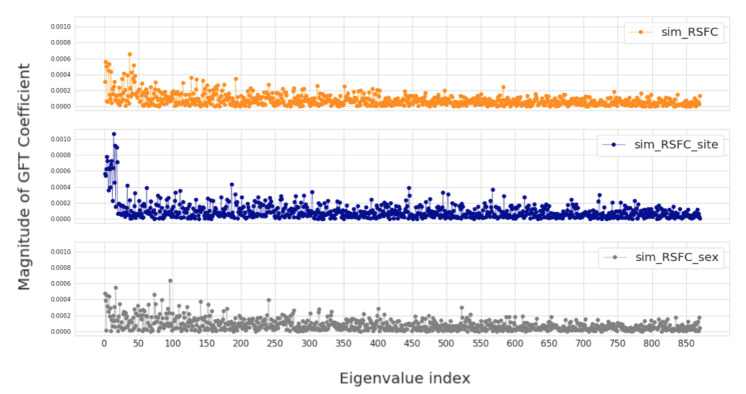
The magnitude of GFT coefficients for graphs which exhibit low-frequency nature. The eigenvalues are sorted in order of the increasing magnitude.

**Figure 5:**
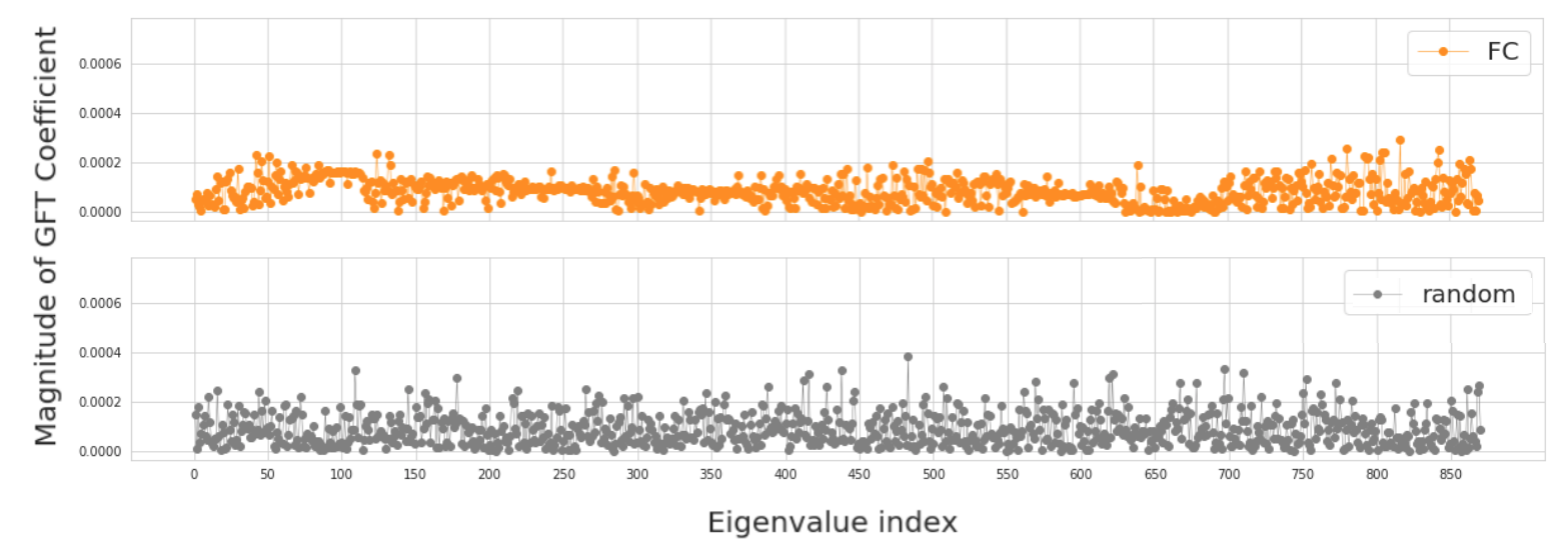
The magnitude of GFT coefficients for graphs which do not exhibit low-frequency nature. The eigenvalues are sorted in order of the increasing magnitude.

In this section, we investigate four groups of constructed population graphs, defined in Section 2.2. Using GFT decomposition, we calculate the magnitude of graph frequency coefficients $\hat{x}$ in order to understand which graph frequency components contribute most to the signal x. Figure 4 and Figure 5 show how the decomposed signals are distributed across each of the following population graphs: *sim_RSFC, sim_RSFC_site, sim_RSFC_sex, FC*, and *random*. We plot the magnitude of GFT coefficients computed for one feature of RSFC-based feature vector x. Clearly, the smoothness of a signal depends on the underlying graph structure. In *sim_RSFC, sim_RSFC_site, sim_RSFC_sex* (Figure 4) the contribution is the highest from low-frequency components. On the other hand, *FC* and *random* graphs are not smooth (Figure 5), as the values of frequency components fluctuate around zero, and the contribution from all frequency components is relatively equivalent and small.

#### 2.4.2. Classification Using Low Frequency Components

In the previous section, we discovered that some of the graphs exhibit a low-frequency nature. Smooth graph signals decay rapidly and can be closely approximated by graph Fourier coefficients [38]. Additionally, taking the first *k* eigenvectors is proven to be efficient for graph clustering purposes [32]. Therefore, by incrementally adding more than one smooth eigenvector, we can improve the performance of graph clustering [39]. We use this fact to test how low and high-frequency components in different graphs contribute towards the accuracy of subject classification. To identify the range of the low best performing graph frequencies, for each graph configuration, we construct a multi-layer feedforward neural network (with two hidden layers of size 512 and 64, separated by ReLU activation function). We train and evaluate the performance of the model on the subject classification task, as follows:

1. Using Equation (4), we filter the graph signal incrementally using the first *k*-frequency components.
2. We train a multi-layer feedforward neural network on the reconstructed features and report test accuracy.

**Figure 6:**
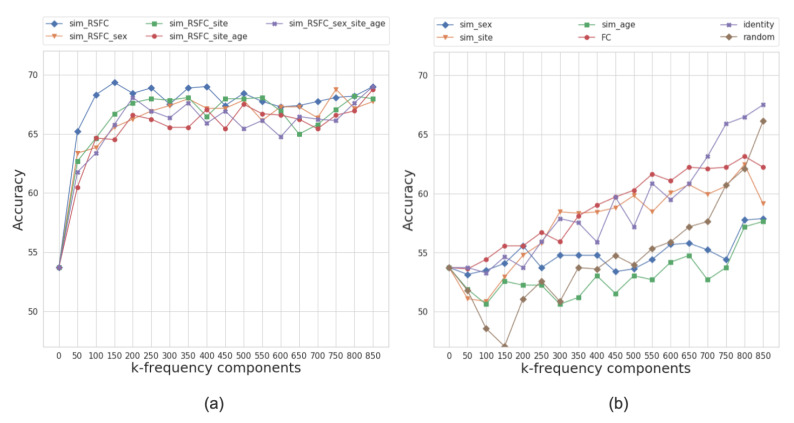
The average accuracy of the classifier over ten folds on frequency-filtered feature vectors for the ABIDE dataset. The plot shows the cumulative contribution of frequency components to the classification accuracy. We split the graphs into two categories based on the performance of models at different frequency regimes, i.e., those that yield higher classification accuracy at (**a**) low or (**b**) high frequencies. The eigenvalues that correspond to k-frequency components are sorted in order of the increasing magnitude.

Figure 6 presents the performance results of the multi-layer feedforward neural network model for different graph configurations. Based on the models’ performance at different frequency regimes, we split the graphs into two categories: those that yield higher classification accuracy at either low or high frequencies.

For the ABIDE dataset, the best performing graph is *sim_RSFC*, i.e., the graph constructed solely using RSFC data. We classify it as a low-frequency graph type since the maximum average accuracy of 69.8% is achieved while only using the first 150 frequency components (first 20% of graph spectrum). The graphs constructed using the combination of RSFC, sex, site, and age (*sim_RSFC_site*, *sim_RSFC_sex_site_age*, *sim_RSFC_sex*, and *sim_RSFC_site_age*) also showed low frequency nature of the graph signal. For all of these graphs, the model achieves the accuracy over 66% on its first 200 frequency components (Figure 6a).

On the other hand, the performance of *identity*, *FC*, *random* graphs, and graphs from *sim_phenotype* group exhibits high-frequency nature, as the model requires seeing all graph frequency components to achieve the best performance (see Figure 6b). It is worth noting that, even after seeing all of the frequency components, the top performance of high-frequency graphs is inferior to the performance of low-frequency graphs. We attribute this to the fact that smooth graphs that use RSFC features capture the underlying structure of the population graphs better than those that do not utilize graph structure or only rely on phenotypic features.

Based on the analysis above, we conclude that the most optimal population graphs are the ones that perform best at low frequencies, i.e., graphs that belong to *sim_RSFC* and *sim_RSFC_phenotype* groups. We assess each of the graphs’ classification performance and select eight best performing low-frequency graphs (one *sim_RSFC* and seven *sim_RSFC_phenotype*) for building a multi-model ensemble.

#### 2.4.3. Population Graph-Based Multi-Model Ensemble for ASD Prediction

**Figure 7:**
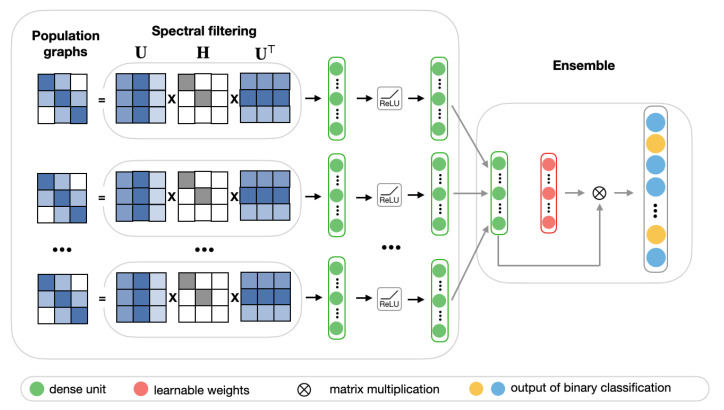
The schematic representation of the ensemble model.

In many cases, the choice of population graph can be problem-dependent and, due to discrepancies of heterogeneous data (such as coming from different sources or having unique features), no population graph can be considered to be entirely accurate. Thus, it is reasonable to consider the performance of multiple population graphs. For this purpose, we propose a multi-model ensemble, which integrates multiple best performing population graphs to yield a better classification performance. The model consists of two stages. Figure 7 shows the schematic representation of the ensemble model.

In the first stage, we select eight best performing low-frequency graphs and, along with RSFC features, pass each of them through the frequency filtering feedforward neural network that is described in Section 2.4.2. Each of these networks can be considered as a learning block, which learns a lower-dimensional representation of input graphs.

In the second stage, we create an ensemble by combining the predictions of each of the eight learning blocks.The ensemble model is implemented as a fully-connected layer with a learnable weighting mechanism. It takes as input n×512 feature vector *p*, which is composed of eight stacked n×64 hidden representations $p_{i}$ from the first stage. For each sample in the test set of size *n*, the final prediction is a binary n×1 vector, with 0 representing a healthy subject and 1 representing a patient diagnosed with ASD. We employ a weighting mechanism for assigning the importance to each individual learning block, corresponding to different input graphs. The classification results of eight models for each subject in the test set are combined by passing them through a softmax layer. For each hidden representation $p_{i}$ from the first stage, the corresponding weight $\alpha_{i}$ is calculated according to the following equation, where *w* is a randomly initialized scoring vector:

(5) $$\alpha_{i}=\frac{\exp\left(w_{i}\right)}{\sum_{j}\exp\left(w_{j}\right)}.$$

After that, the weighted sum of the individual prediction blocks $H=\sum_{i}\alpha_{i}p_{i}$ is passed into the sigmoid layer:

(6) $$\hat{y}=\mathrm{sigmoid}\left(WH+b\right),$$

where *W* and *b* are weight and bias matrices of fully-connected layer, respectively. Finally, the loss is calculated while using the cross-entropy error over the labeled samples:

(7) $$L=-\sum_{c}Y_{c}\log\left({\hat{Y}}_{c}\right)$$

where $Y_{c}$ and ${\hat{Y}}_{c}$ is the actual and the predicted labels.

## 3. Results

In this section, we evaluate the performance of our two-stage population graph-based ensemble model. First, we describe the training configuration and baselines that were used for comparison. Subsequently, we present the evaluation and discuss the competitiveness of our proposed model as compared to the recently published works.

### 3.1. Training Setup

Because od the limited size of the ABIDE dataset and the sake of fair comparison with state-of-the-art methods, we used ten-fold stratified cross-validation, with the stratification criterion being an equal allocation of the class labels: patients with ASD and healthy subjects. The hyperparameters for each graph-based model in the first stage (the number of layers, the learning rate, dropout rate, etc.) were tuned using Optuna hyperparameter optimization framework [40]. We trained a graph-based model on 200 epochs with Adam [41] optimizer and learning rate = 0.01. We fixed graph filtering frequency threshold to k=200. All of the models were developed while using open-source machine learning library PyTorch [42] and trained on NVIDIA GeForce GTX TITAN X GPU with 12GB memory size and CPU Intel(R) Core(TM) i7-7700K CPU @ 4.20GHz.

### 3.2. The Baselines Used for Comparison

We compare our model with the following single-model methods and with multi-model ensemble approaches that were developed for brain imaging datasets:

- **Kernel regression** [43] is a classical machine learning algorithm used by the authors for predicting subject phenotypes from RSFC. It achieved comparable performance to several deep neural networks on several brain imaging datasets. We include this model as a baseline for classifying ASD while using the ABIDE dataset.
- **DNN** [15] utilized RSFC features to build an autoencoder-based deep neural network for classifying ASD. The authors used a subset of 964 subjects from the ABIDE dataset and achieved a classification accuracy of 70%.
- **CNN** [2] proposed a convolutional neural network architecture in order to classify ASD patients and control subjects while using RSFC. The authors reported the classification accuracy of 70.22% on a subset of 871 subjects from the ABIDE dataset.
- **ASD-DiagNet** [16] increased the computational speed of autoencoder based DNN [15] by introducing a hybrid learning procedure. Similarly, only RSFC features were used as the input to the model.
- **GCN** [4] introduced a graph neural network that allowed incorporating both RSFC and phenotypic features. The authors tested the model on a set of 871 subjects from the ABIDE dataset.
- **Ensemble_mv** [44] utilized multiple discriminative restricted Boltzmann machines (DRBM) to classify 263 subjects from the ABIDE dataset. The majority voting strategy was used for combining the prediction outputs of individual DRBMs.
- **Ensemble_bootstrap** [31] proposed a bootstrapping approach by generating twenty randomized graphs from the initial population graph. Each randomized graph was passed through a graph neural network. The final output of the ensemble was determined by averaging the probability estimates of each of the networks.

### 3.3. Comparison with the Baselines

This section presents the accuracy results of all baselines and ablation studies while using stratified ten-fold cross-validation (see Table 2). We compare our approach with single-model state-of-the-art methods presented at the top of the Table 2. These models include Kernel regression [43], deep learning-based frameworks DNN [15], convolutional neural networks CNN [2], and ASD-DiagNet [16], which only use RSFC features as an input, and the graph-based GCN model [4] which uses both RSFC and phenotypic features. In addition to single-model approaches, we compare our method with recent multi-model ensembles on ABIDE dataset, which are Ensemble_bootstrap [31] and majority voting-based ensemble method Ensemble_mv used in [44]. We also perform several ablation studies for single- and multi-model cases to test the contribution of each component of the model. To provide a fair comparison, we ran all of the baseline models on the same subset of subjects used in [4], consisting of 403 patients with ASD and 468 healthy individuals.

Additionally, we introduce three baselines: *No graph*, *Random graph random*, and fully-connected *FC graph* to evaluate the meaningfulness of the proposed edge construction method. *No graph* baseline evaluates the contribution of fMRI features alone, regardless of the underlying population graph. This means that the underlying graph becomes completely disconnected, i.e., no two nodes are connected. In *Graph random*, the edges are randomly rewired. *FC graph* baseline is implemented, so that all of the connections in a population graph are equal to one.

Based on the results, we conclude that our ensemble model significantly outperforms baseline methods, providing a performance gain in accuracy between 2.91% to 7.35%. For example, our model’s classification accuracy on the ABIDE dataset is significantly higher when compared to the top results that were achieved in the single-model case by CNN [2], i.e., 73.13% vs. 70.22%. Similarly, among multi-model ensembles, our approach outperforms both Ensemble_bootstrap [31] and Ensemble_mv [45] (73.13% vs. 65.78% and 68.19%, respectively). In addition, our model achieves the highest average AUC (0.75) and F-score (0.75) across ten folds among all of the baselines.

When compared to a single-model graph-based baseline GCN [4], our multi-model ensemble achieves more than five times boost in speed. The boost in computational time is attributed to signal filtering, which eliminates the need for performing multiple graph convolution operations on the input matrix that was used in [4]. This is particularly important if we consider datasets of a large scale in the future. Moreover, our model performed 80 times faster than the multi-model graph-based ensemble approach that was proposed by [31]. In addition, our model achieves the best performance in terms of accuracy and AUC as compared to *No graph*, *Random graph random*, and *FC graph* baselines, which justifies the usefulness of the constructed population graphs. Consistently higher accuracy of our model over other baselines shows its robustness of capturing the population graph structure and extracting useful features.

**Figure 8:**
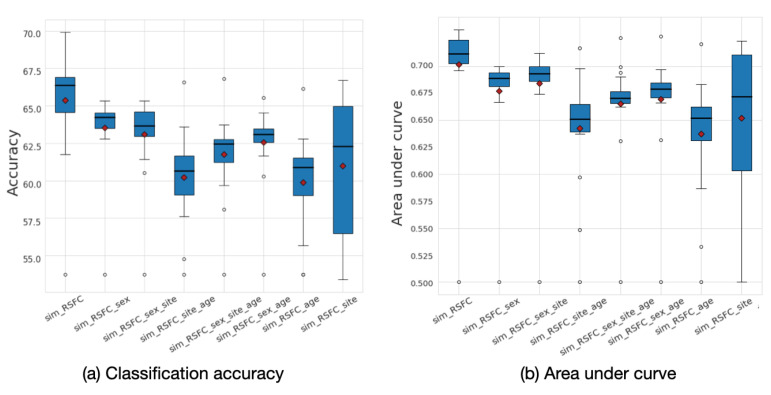
(**a**) Classification accuracy and (**b**) area under curve (AUC) results for each of the eight input graphs run separately for the ABIDE dataset. The mean values are shown as red diamonds.

The analysis of the underlying graph property allowed for us to choose the best performing low-frequency graphs and, consequently, build a framework that outperforms the baselines. To explore the effect of graph frequency filtering, we present the accuracy results that were calculated for different frequency thresholds for each of the eight input graphs in Figure 8. The results demonstrate that the most informative graphs are those that were constructed using RSFC data, which, due to graph construction design, are low-frequency by nature. By separately analyzing the performance of each of the eight input graphs, we can see that the *sim_RSFC* graph alone achieves relatively higher performance than other graphs, with the maximum accuracy of 69.8%, which is on par with the existing baselines. Introducing graph frequency filtering improves the ensemble’s overall performance by 1.04% when compared to the Ensemble_no_gsp model, in which no filtering was used.

By definition of the edge assigning function, the population graph is being constructed in such a way that neighboring nodes have similar features (based on age, sex, site, and RSFC). If we look from eigendecomposition, then eigenvectors will be the smoothest (those that correspond to low eigenvalues) in the case when similar features lie on the neighboring nodes. Thus, we are interested in the case when graph definition reinforces the smoothness property. In a graph with a smooth signal, the unknown subject will have a higher chance of being labeled healthy if connected to many healthy subjects. When we incorporate other non-imaging features to define edges in the graph, we want to reinforce the signal smoothness. High-frequency components are more likely to increase the contribution of signal noise that arises from edges connecting nodes with dissimilar features. Therefore, by analyzing the performance of the model on low-frequency components, we can distinguish between features that contribute towards the signal smoothness and improve the performance, and features that reinforce edge weights between dissimilar nodes and, thus, introduce the noise.

The ensemble approach that we proposed in this work further improves the accuracy by setting a frequency filtering threshold and efficiently combining weighted contributions from each graph. Our choice of integrating multiple graph-based models into an ensemble was motivated by comparison with several ensembling schemes. Because simple averaging [31] of the results from individual models and majority voting algorithm [44] did not yield superior performance, we proposed an end-to-end deep neural network-based ensemble model that uses the weighting mechanism to assign different levels of importance for each model’s prediction.

### 3.4. Sensitivity to Frequency Filtering

**Figure 9:**
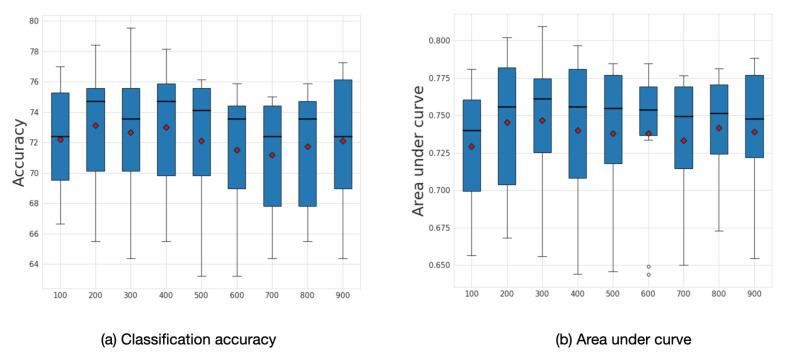
Sensitivity analysis for *k*. Average accuracy and AUC results with respect to the frequency filtering threshold *k*. The mean values are shown as a red diamonds.

We explored the influence of frequency filtering threshold *k*, which we varied from 100 to 900. We found that lower frequency filtering thresholds resulted in improved performance. Figure 9 illustrates the box plot for average accuracy and area under curve (AUC) calculated across ten folds on the ABIDE dataset. The model’s highest performance is achieved within the range of frequency filtering threshold between 200 and 400. The best average accuracy corresponds to the case when the frequency filtering threshold equals 200 with an accuracy of 73.13% and AUC 0.75.

Based on the analysis, we build a multi-model classification ensemble that focuses on low-frequency population graphs. The results show that the proposed ensemble improves the prediction performance when compared to the single-model case by 3.33%, and presents robustness to the choice of the input graphs.

In this work, the signal filtering parameter *k* is fixed to be the same for all of the selected graphs. Based on our model’s performance on different *k*-frequency components, we specify *k* to be 200. Determining the optimal *k* for each graph configuration can lead to improved performance of the ensemble overall and it is yet to be explored in future work. For example, an attention mechanism can be explored to learn the best performing *k* for each graph configuration. Another potential future work can focus on learning the population graph structure, so that the input data form graph signals with smooth variations [36,46,47,48].

## 4. Conclusions

In this paper, we proposed a graph-based multi-model ensemble for diagnosing Autism Spectrum Disorder. While earlier works evaluated the importance of non-imaging features that were based on classification accuracy, we instead analyzed how non-imaging features affect the population graph’s smoothness property. Specifically, we analyzed the population graph structure resulting from a different choice of edge defining function. As far as we know, this is the first work to perform an extensive comparison of multiple graph configurations while using GSP on the ABIDE dataset. The results show that different combinations of RSFC and phenotypic features result in a different graph structure, affecting the accuracy of the prediction. In this paper, we address this issue by integrating multiple graph-based prediction models into an ensemble. First, using graph filtering, we selected the best performing graphs by removing the contribution from high-frequency components. Second, we combine multiple graph-based models in order to construct a more powerful ensemble. We performed extensive comparisons of our model with several state-of-the-art baselines. The results demonstrate that the proposed ensemble model outperforms other baselines in terms of accuracy, area under curve, and F-score.

## Figures and Tables

**Table 1:** The statistics of the Autism Brain Imaging Data Exchange (ABIDE) dataset that was used in this work.

Total subjects	871
Patients/Healthy controls	403/468
Female/Male	144/727
Age range	6–58
Age mean	16.94
Imaging sites	17

**Table 2:** Classification results of baselines and ablation studies. The results for Kernel Regression [43], GCN [4] and Ensemble_bootstrap [31] were calculated using our implementation. The performance gain of our model with respect to the given baselines is in terms of accuracy. The bold font shows the best performance. n.a stands for not available.

	Accuracy (%)	AUC	Sensitivity	Specificity	F-Score	Time (s)	Performance Gain (%)
Kernel Regression [43]	67.50 ± 5.51	0.73 ± 0.06	0.79 ± 0.06	0.53 ± 0.07	0.72 ± 0.05	4	5.63
GCN [4]	69.11 ± 4.38	0.73 ± 0.05	0.75 ± 0.06	0.57 ± 0.05	0.70 ± 0.04	892	4.02
CNN[2]	70.22 ± 8.55	0.74 ± 0.01	0.77 ± 0.01	0.61 ± 0.01	0.73 ± n.a	45000	2.91
DNN [15]	70.00 ± n.a	0.74 ± n.a	0.74 ± n.a	0.63 ± n.a	n.a	118356	3.13
ASD-DiagNet [16]	69.59 ± 4.90	0.67 ± 0.23	0.65 ± 0.06	**0.72 ± 0.07**	0.66 ± 0.03	1452	3.54
No graph	67.50 ± 4.42	0.74 ± 0.05	0.76 ± 0.33	0.31 ± 0.40	0.60 ± 0.14	62	7.34
Random graph	66.12 ± 4.97	0.74 ± 0.05	0.86 ± 0.10	0.25 ± 0.17	0.68 ± 0.02	63	8.15
FC graph	62.23 ± 5.92	0.66 ± 0.04	**0.86** ± **0.07**	0.34 ± 0.10	0.71 ± 0.02	53	11.81
Ensemble_mv [45]	65.78 ± 4.44	0.72 ± 0.04	0.68 ± 0.07	0.63 ± 0.09	0.68 ± 0.04	255	7.35
Ensemble_bootsrap [31]	68.19 ± 3.82	0.73 ± 0.04	0.75 ± 0.05	0.59 ± 0.05	0.72 ± 0.03	16,138	4.94
Ensemble_no_gsp (ours)	72.09 ± 4.30	0.74 ± 0.04	0.74 ± 0.13	0.69 ± 0.11	0.74 ± 0.06	140	1.04
Ensemble_gsp (ours)	**73.13** ± **4.31**	**0.75** ± **0.04**	0.76 ± 0.07	0.69 ± 0.05	**0.75** ± **0.04**	276	–
